# A Deep-Learning-Based Health Indicator Constructor Using Kullback–Leibler Divergence for Predicting the Remaining Useful Life of Concrete Structures

**DOI:** 10.3390/s22103687

**Published:** 2022-05-12

**Authors:** Tuan-Khai Nguyen, Zahoor Ahmad, Jong-Myon Kim

**Affiliations:** Department of Electrical, Electronics and Computer Engineering, University of Ulsan, Ulsan 44610, Korea; khaint@mail.ulsan.ac.kr (T.-K.N.); zahooruou@mail.ulsan.ac.kr (Z.A.)

**Keywords:** acoustic emission, deep neural network, concrete structures, health indicator, Kullback–Leibler divergence, remaining useful life, stacked autoencoder

## Abstract

This paper proposes a new technique for the construction of a concrete-beam health indicator based on the Kullback–Leibler divergence (KLD) and deep learning. Health indicator (HI) construction is a vital part of remaining useful lifetime (RUL) approaches for monitoring the health of concrete structures. Through the construction of a HI, the deterioration process can be processed and portrayed so that it can be forwarded to a prediction module for RUL prognosis. The degradation progression and failure can be identified by predicting the RUL based on the situation of the current specimen; as a result, maintenance can be planned to reduce safety risks, reduce financial costs, and prolong the specimen’s useful lifetime. The portrayal of deterioration through HI construction from raw acoustic emission (AE) data is performed using a deep neural network (DNN), whose parameters are obtained by pretraining and fine tuning using a stack autoencoder (SAE). Kullback–Leibler divergence, which is calculated between a reference normal-conditioned signal and a current unknown signal, was used to represent the deterioration process of concrete structures, which has not been investigated for the concrete beams so far. The DNN-based constructor then learns to generate HI from raw data with KLD values as the training label. The HI construction result was evaluated with run-to-fail test data of concrete specimens with two measurements: fitness analysis of the construction result and RUL prognosis. The results confirm the reliability of KLD in portraying the deterioration process, showing a large improvement in comparison to other methods. In addition, this method requires no adept knowledge of the nature of the AE or the system fault, which is more favorable than model-based approaches where this level of expertise is compulsory. Furthermore, AE offers in-service monitoring, allowing the RUL prognosis task to be performed without disrupting the specimen’s work.

## 1. Introduction

With the easy access and high durability, concrete structures have become a ubiquitary sight in recent decades. Along with their presence everywhere, there is the inevitable need for a maintenance plan in order to ensure in-service safety and to prolong the lifetime of concrete structures. Numerous studies [1,2,3,4,5,6,7,8,9,10] have been conducted by laboratories, companies, and individuals racing to identify solutions regarding the performance amelioration of structural health monitoring (SHM). By employing an effective monitoring scheme, it is possible to provide the user with more insight into the in-service system/structure, avoid near-future failures, and lessen downtime by a significant amount. 

Among SHM topics, the remaining useful lifetime (RUL) prognosis is one of the most concerned problems, in which a solution is provided to predict how long a structure can be used until it is permanently out-of-service because of a failure(s). By predicting the future state of the structure, a user can approximate the time when a failure might occur, thus adjusting the usage and preparing a maintenance plan accordingly in advance. The most recent studies [11,12,13,14,15,16] regarding this topic focus on the development of an autonomous system that is capable of extracting feature(s) from data and constructing indicators based on these extracted features. A common scheme for this approach is as follows: initially, sensors are deployed, which are then utilized to collect and send the real-time data to the central brain where data processing and health indicator construction occur.

As nondestructive methods for SHM have become dominant in recent years, different methods have been investigated for concrete SHM such as ultrasonic [5,17,18], vibration [19,20], image processing [3,21,22], and acoustic emission (AE) [4,12,23,24,25,26,27,28,29,30,31]. Even though ultrasonic testing can detect internal defects and their sizes, it is susceptible to complicated part geometry and certain materials, such as austenitic steel, which can mask defects by causing attenuation. Concerning vibration techniques, they are restricted by their limited band of frequency. In addition, although image processing techniques offer the most facile setup among all mentioned methods, they are only capable of detecting failures on the surface. In comparison to these nondestructive methods, AE techniques, which study and exploit the release of internal elastic energy by a discontinuity appearance, offer a nondirectional means of monitoring capable of achieving in-service testing without downtime [32]. A single test of AE can allow users to track the specimen’s deterioration process dynamically, while highlighting the severity of the damage. The drawback of this method is that AE can only be utilized to detect the occurrence of new discontinuities, not existing ones. However, as the purpose of the AE test is to detect novelties to the current state of the specimen, this drawback can be considered insignificant in comparison to the benefits that it provides. In this study, we focus on employing a technique using AE sensors.

The data collected by AE sensors are then analyzed for health indicator (HI) construction. The approaches of HI construction for RUL prediction, similar to the prognosis and health management framework in general, can be roughly classified as model-based or data-based. The methods following the first category focus on real-life process imitation, which is established via mathematical means. Given the drawback of being increasingly problematic as the model becomes more complex, it is less favorable than the data-based approach, in which the interested patterns are derived from available data even in the absence of knowledge about the nature of the system or the fault. Therefore, the data-based approach is the main priority of this study. Further discussion on HI construction can be found in Section 2.

To ensure the reliability of the constructed HI, an evaluation is needed. According to a previous survey [33], the evaluation of HI can be performed by two approaches: one concerning the fitness analysis of the construction result and the other considering the RUL prognosis performance. Further discussion on the HI construction framework and evaluation is continued in Section 2.

**Figure 1:**
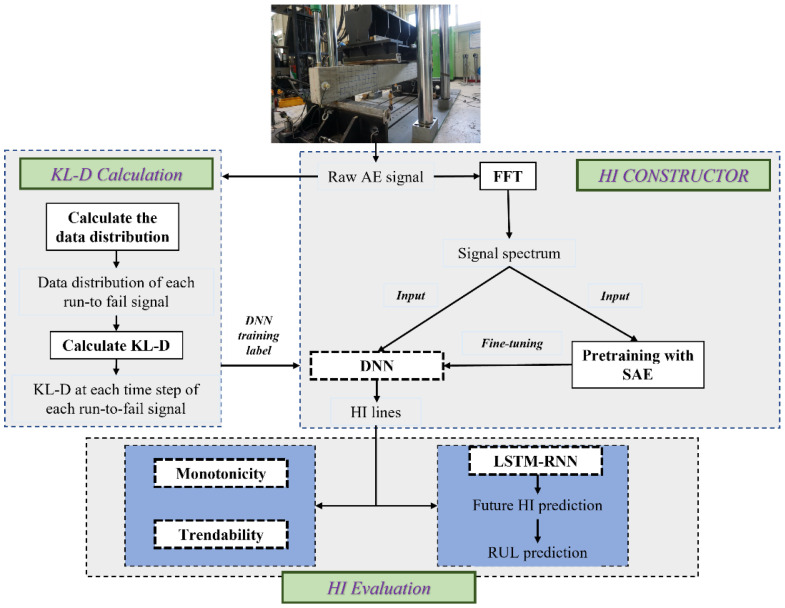
Flowchart of the HI construction framework and evaluation process.

The HI construction and evaluation is presented in Figure 1:

Initially, the raw data are collected from the specimen throughout its deterioration process. It is then utilized for the calculation of Kullback–Leibler divergence (KLD) at each time step of each run-to-fail signal. Afterward, the raw signal is fed to the HI constructor, where its spectrum is computed and used to reduce computation complexity. The stacked auto-encoder (SAE) takes the spectrum as input for the pretraining process, which is subsequently fine-tuned into the deep neural network (DNN) to construct the HI lines with the calculated KLD as the training label. Finally, the evaluation of HI is performed using both intrinsic measures and RUL prediction to test the proposed method’s reliability.

In summary, this study proposes a data-based approach for HI construction based on KLD, for which the expertise concerning concrete material and the nature of the fracturing process is not required. The constructed HI, through its ability to accurately describe the deterioration process of concrete structures, can be utilized for RUL prognosis and can allow users to plan maintenance prior to possible structural failures in the near future. The following contributions are proposed:

1. A novel HI construction process is proposed using KLD to describe the state of the concrete specimen during its lifetime directly from raw AE data, which has not been previously investigated to the extent of the authors’ knowledge.
2. Evaluation of the proposed HI construction using both fitness analysis and RUL prediction.

The arrangement of the following sections is as follows: Section 2 discusses the general HI construction framework and evaluation; the process of HI construction is provided in detail in Section 3; Section 4 describes the experimental setup and HI evaluation using the two discussed methods; finally, Section 5 provides a conclusion, along with a discussion of future research possibilities.

## 2. The General Health Indicator Construction Framework and Evaluation

HI construction is an essential part of the prognosis to portray the timeline of deterioration. HI shows the condition in which the specimen under investigation is. By analyzing the current HI, it is possible to predict future values and their timing, thus allowing the RUL to be estimated. Over the years, numerous studies have proposed different approaches to build a HI, especially in more recent studies [34,35,36], and in summary, this process can be generally divided into two steps: (1) calculation of the HI-constructing factor(s) and (2) HI construction from the calculated factor(s).

The calculation of the HI-constructing factor(s) (also known as features) is often performed either in the time, frequency, or time–frequency domain. The time domain approaches [27,28,37,38] generally offer a fast and simple solution that can be widely applicable to systems and fault types. They often include statistical computation and impulse analysis. These methods, however, are susceptible to interferences, which are typically inevitable in real-life applications. Therefore, pre-processing techniques are necessary to minimize performance degradation. Frequency domain solutions [39,40] explore anomalies in the system with prior information of the fault characteristic frequencies already known. They are often adapted in model-based methods and can offer high efficiency; however, they are not widely applicable. The approaches following a time–frequency domain solution [41] are the most powerful among these three and can be highly robust. Their downside is that such powerful methods often require high computational capability and experience concerning information extraction.

Afterward, the second step is to build the HI constructor. As briefed in the previous section, the construction of a HI can be roughly divided into two categories: model-based and data-based. Model-based methods focus on generating a mathematical representation that mimics a real-life process. The development of a HI in these studies requires expertise, knowledge about the system’s behavior, the nature of the faults, and the HI-constructing factors. Following this approach, the studies in [11,26,40,41] investigated the faults with respect to fault-related frequencies, which indicates that the comprehension of the system and fault nature is necessary. Another study in [42] proposed an effective HI construction method with features manually chosen based on relevance. The aforementioned research and many others have been carried out with expertise in signal processing, and system and fault behavior, which can be troublesome to obtain in more sophisticated scenarios. Moreover, they cannot be adapted to a variation in systems, due to their construction imitating certain real-life processes. Unlike model-based approaches, data-based solutions focus on the nature of the data itself, with less concern for the system or fault nature. Due to their lower complexity and wider application toward different systems and faults, data-based methods have been more favorable in recent years, especially with the rise of artificial intelligence [43]. Notable mentions in this category are statistical projection [39,44,45], deep learning models [12,46,47,48], and evolutionary computation [33,49], etc. Different studies following these methods have achieved promising results in HI construction for the RUL prognosis task.

To verify the constructed HI, the evaluation process must be performed with suitable metrics. Concerning the HI construction for RUL prognosis, the evaluation can be generally divided into two categories: the investigation of the HI’s intrinsic nature from the construction result (fitness analysis) and the performance of HI in the RUL prognosis tasks. Fitness analysis is often performed with the following metrics: monotonicity [12,41] (measurement of the monotonic trend in HI), trendability [12,41] (the correlation of HI and time), and scale similarity [12,41,48] (similarity of HI ranges), etc. The purpose of this type of evaluation is to self-reflect the HI properties via low computational complexity without concern of the prognosis task. Furthermore, the second category evaluates HI by its performance in RUL prognosis. This indirect assessment can be performed through the mean absolute error, mean square error, mean absolute deviation, etc. In comparison to the first category, it provides more information about the prognosis task as a whole; however, it requires more computational complexity because the whole prognosis block is added. The proposed method is verified in both categories, with monotonicity and trendability in the first and mean absolute error in the second evaluation.

## 3. The Proposed Health Indicator Construction

**Figure 2:**
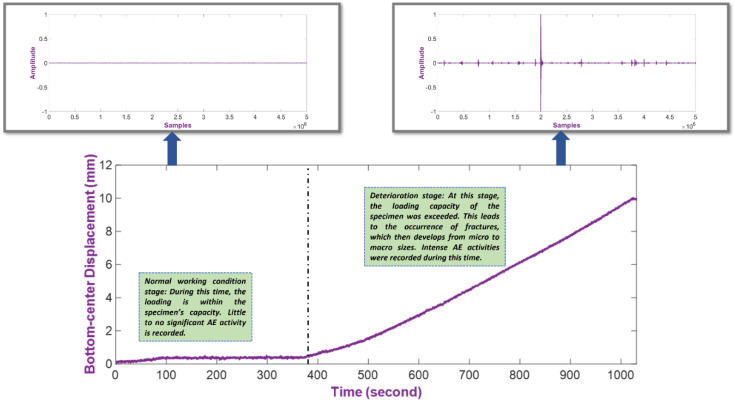
Vertical displacement of the specimen and signal difference in two loading stages.

KLD is used to construct the HI to investigate how different an unknown signal is to a known normal condition. As the deterioration progresses during the loading test, it can be expected that the difference of the unknown signal to the reference one grows with the intensity of the AE activity. For that reason, this difference is suitable for the portrayal of the deterioration process from the run-to-failure signals. Figure 2 shows a loading test’s stages of deterioration and highlights the difference between a reference signal to a signal in which significant AE activities are recorded. The detailed experimental setup and data description are discussed in Section 4. As shown in Figure 2, the vertical displacement of the specimen grows steadily during the deterioration stage, which indicates continuous damage being sustained; therefore, it is also evident that significant AE activities occur during this period.

In this study, the signal is segmented into one-second blocks (time steps) for the computations. The HI construction process initiates with the calculation of each run-to-fail signal’s probability distribution. A signal’s values are divided into segments of 1 × 10^−3^ width ranging from its minimum value to its maxima, at each of which the probability density function (PDF) of the signal is computed. The result of this computation is later utilized for the KLD calculation.

The divergence of two probability measures was originally defined by Harold Jeffreys [50] in a symmetrized form (directed divergences, also known as the relative entropies in each direction), which is now referred to as the Jeffrey Divergence. Later in the 1950s, Solomon Kullback and Richard Leibler [51] proposed exploring the mean information discrimination between two hypotheses by their according probability measures using relative entropy in an asymmetric manner. This concept later came to be known as the Kullback–Leibler Divergence. Its first context was developed for information theory and later widely adapted in optimization tasks of machine learning. The value of KLD falls within the range of 0 to 1, which indicates no difference and maximum divergence, respectively. Assuming two probability distributions $P\left(x_{i}\right)$ and $Q\left(x_{i}\right)$ from the reference normal condition and unknown signal, their KLD calculation is as follows:

(1) $$\mathrm{KLD}\left(P,Q\right)=\sum_{i=1}^{N}P\left(x_{i}\right)log\frac{P\left(x_{i}\right)}{Q\left(x_{i}\right)},$$

It is assumed that all of the run-to-fail signals start at a normal state with little to no significant AE activity recorded during this period. Therefore, a reference normal-conditioned signal can be arbitrarily selected from the very start of the recording.

With the KLD values, the process continues with the establishment of the DNN-based constructor, which outputs HI lines from the inputted signal spectrum. Initially, the signal spectrum is calculated using the fast Fourier transform and then equally separated into 2 × 10^3^ size bands, whose energy can be approximated using the root mean square. By doing such an action, the computation capacity can be reduced for the following operations. Afterward, the pre-training of DNN commences as SAE is fed with the obtained vector of size 2 × 10^3^. The SAE consists of an encoder and a decoder, both comprising three dense layers. The encoder processes the data through layers of diminishing sizes (1000-200-10) in the encoder and then layers of increasing size (200-1000-2000) in the decoder. Xavier initialization and an exponential linear unit activation are harnessed for the encoder. In addition, dropout layers of rate 0.1 are appended before the dense layers for the amelioration of the SAE’s regularization. The training of SAE is executed with the gradient-descent parameter update, Adam optimization, and a 0.1 fraction of masked zeros, along with an unlabeled signal spectrum as both the input and output. Noise is added to the training data, which forces the reconstruction of the noisy input, thus improving the learnt features’ robustness.

Once the SAE’s training concludes, fine-tuning of the DNN follows as the encoder’s layers are utilized as the DNN’s hidden layers, on top of which a logistic regression layer is placed. The calculated KLD value, which falls between the range of [0, 1], is utilized as the output for the DNN training, along with the input of the signal spectrum. In addition to freezing the reused layers for learning ability preservation, early stopping and checkpoint are also used to achieve better parameters. Similar to the KLD, the constructed HI is also within [0, 1], which indicates the damage the specimen has sustained from the least to the most in the severity scale.

## 4. Experimental Setup and Evaluation

### 4.1. Experimental Setup

**Figure 3:**
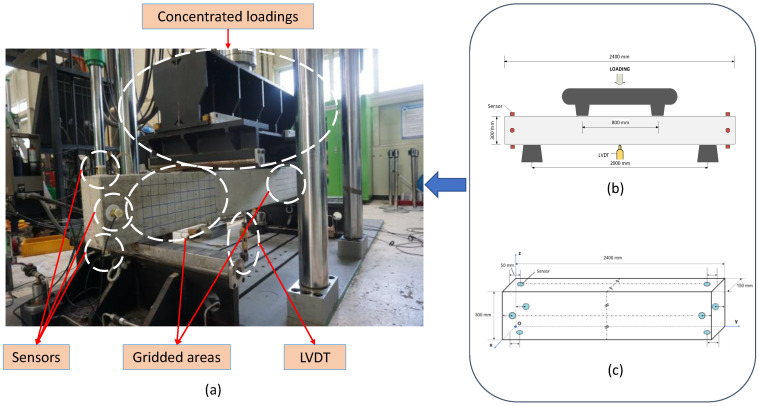
Experimental setup of the four-point bending test on reinforced concrete beam: (**a**) pictorial of the specimen under test, (**b**) schematic of the four-point bending arrangement, and (**c**) placement of sensors on the specimen.

To evaluate the reliability of the proposed method, data were collected from reinforced concrete beams, which were constructed and installed for the four-point bending test scenario. Each specimen was identically produced with concrete material having a compressive strength of 24 MPa, D16 (SD400) steel rebar, and gridded in 50 × 50 mm^2^ squares for better visualization of deterioration monitoring. The AE data were acquired at 5 MHz from eight R3I-AST sensors placed around both ends of a specimen. The detailed four-point bending test setup and sensor placement are shown in Figure 3.

**Figure 4:**
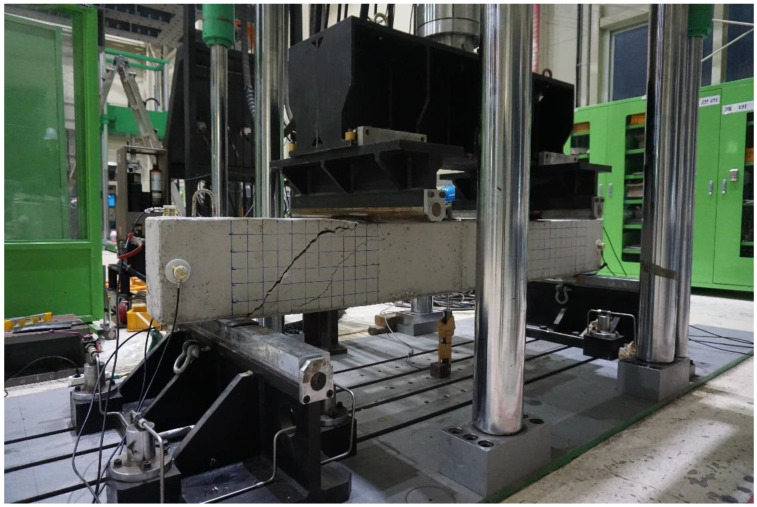
Damage sustained by a specimen during the test.

Each specimen was subject to two-point concentrated loading. The loading points were placed 400 mm from the left and right of the specimen’s median, and the stress was applied at a speed of 1 mm/s. In addition, a linear variable differential transformer (LDVT) was set underneath the specimen at its center for vertical displacement measurement. This is an alternate way to track the damage sustained other than the visual monitoring of the specimen’s surface fractures. During the test, the specimens were loaded until the damage was high enough that maintenance was not efficient anymore; however, total collapse was prohibited from happening for the safety of the observers that included our team members and construction field specialists. Figure 4 shows the damage sustained by a specimen during the test:

Data from three tests, α, β, and γ, were harnessed for this study. Each test was recorded with eight AE sensors with a total of 24 run-to-fail signals. For each test, signals from one side (recorded from four out of eight sensors) were used for the training processes, and the rest for testing. The durations of the tests were 600, 650, and 620 s, respectively.

### 4.2. Evaluation and Discussion

**Figure 5:**
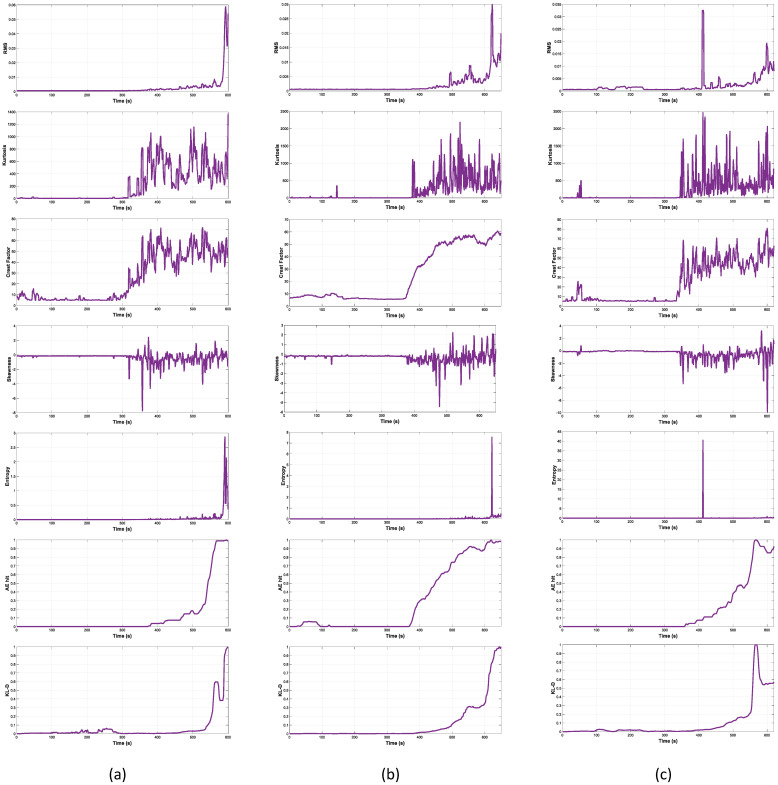
Visualization of the proposed and other HI-constructing factors (RMS, Kurtosis, Crest Factor, Skewness, and AE hit) from sensor 5 of reinforced concrete beam: (**a**) α, (**b**) β, and (**c**) γ.

As aforementioned, the reliability of our proposed method was verified by two evaluations: fitness analysis of the construction result and RUL prognosis performance. In this section, these evaluations are demonstrated in comparison with other methods. The proposed and other HI-constructing factors are visualized in Figure 5.

In order to analyze the HI construction result, trendability and monotonicity were utilized as the evaluation metrics. In the prognosis tasks, trendability can be perceived as the indicator of a feature’s variation with regard to time. High trendability can be witnessed in linear functions, whereas a constant function represents the minimum trendability. The resulting line of reliable HI construction is expected to be trendable, especially as the specimen progresses closer to its failure. In our study, this metric is computed as:

(2) $$\mathrm{Trendability}=\frac{N\sum xt-\sum xt}{\sqrt{\left[N\sum x^{2}-{\left(\sum x\right)}^{2}\right]\left[N\sum t^{2}-{\left(\sum t\right)}^{2}\right]}},$$

where *N* is the observation number, and x and t are the feature and time index, respectively.

In addition, monotonicity characterizes the underlying decreasing or increasing trend. Its value is in the range of [0, 1], which indicates a higher level of monotonicity as it rises. A good HI result is expected to not possess a low level of monotonicity. The computation of this metric can be performed as follows:

(3) Monotonicity=absolute(nddx>0− nddx<0N−1),

where *N* is the observation number.

To verify the fitness of the KLD-based HI, a comparison was made with various HI-constructing factors. The results based on the two metrics are shown in Table 1.

From what can be witnessed in the table, the AE-hit-based HI and KLD-based HI are comparably outstanding from others in terms of the two concerned metrics. Most of the other HI-constructing factors can show a middle to high trendability (especially Crest Factor with trendability value of 0.7416, which is more than both AE-hit and KLD); however, they are lacking in terms of monotonicity. Such a behavior indicates a poor characterization of the underlying increasing trend toward the end of the specimen’s HI. The KLD HI presents a high level of monotonicity and trendability, especially toward the end of its useful lifetime, as shown in Figure 5. This is an implication that the use of KLD is suitable for the portrayal of a concrete structure’s deterioration process.

In addition to the fitness analysis of the construction result, the HI was also evaluated by its performance concerning RUL prognosis. A long short-term memory recurrent neural network (LSTM-RNN) [28] was chosen for this purpose. Because each run-to-fail signal is a sequence of values at the time steps, it can be deemed as a univariate time series. The LSTM-RNN takes in the signals in form of a 50-sample sliding window, which moves one sample at a time, and predicts the 51st sample. By performing such an action, the model is forced to predict not just the final value, but at each window of the signal. In addition, it also enhances the training speed and stabilization by enabling more error gradient. This model contains an input layer, two hidden size-of-20 LSTM layers, and a dense output layer using a linear activation function. Similar to DNN training, early stopping and checkpoint techniques are also used here.

**Figure 6:**
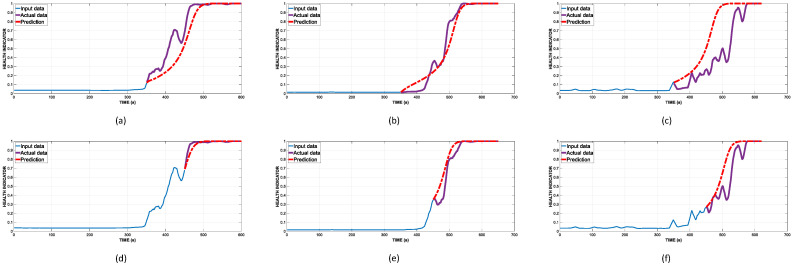
Prediction of future HI from the three tests at two major time steps. (**a**) Reinforced concrete beam α at the 350th time step. (**b**) Reinforced concrete beam β at the 350th time step. (**c**) Reinforced concrete beam γ tests at the 350th time step. (**d**) Reinforced concrete beam α at the 450th time step. (**e**) Reinforced concrete beam β at the 450th time step. (**f**) Reinforced concrete beam γ at the 450th time step.

The importance of RUL prognosis increases with time. During the normal working stage with no damage sustained yet, it is inessential to make a prediction. From the moment the specimen enters its deterioration stage, the importance of RUL prognosis grows significantly due to the need of maintenance planning. As a result, we chose two major time steps at 350 and 450 to perform the prediction, which are the first appearance of minor and major fractures, respectively. Figure 6 displays the HI prediction from the three tests at these time steps.

In our study, the useful lifetime expiration is marked at the first occurrence of 0.95 along the HI line; therefore, the RUL can be calculated as follows:

(4) $$\mathrm{RUL}=T_{0.95}-T,$$

where $T_{0.95}$ is the time step when the HI reaches 0.95 and T is the time step at which RUL prognosis is being performed. Afterward, an error computation is needed to determine how much the RUL prediction differs from the actual RUL:

(5) $$\mathrm{Absolute}\;\mathrm{error}=\mathrm{absolute}\left(RUL_{predicted}-RUL_{actual}\right),$$

where $RUL_{predicted}$ is the predicted RUL and $RUL_{actual}$ is the actual RUL. While this measurement is capable of demonstrating the distance between the predicted and actual RUL, it cannot show which of the predicted and the actual useful life expiration comes first.

The summarized prediction result at two time steps is shown in Table 2. In addition to the plots shown in Figure 6, it can be seen that the prediction closely follows the underlying trend of the actual HI, especially with the first two tests. The KLD based HI offers better prediction of the RUL in comparison to AE-hit based methods, with or without anomalous hits removal (AHR), by a significant margin. From the prediction performed at the 350th time step, the average errors are 28, 28, and 36, respectively, in the three tests. On the second prediction, the result is remarkably closer because the test errors are, on average, 11, 18, and 16. It is also worth noting that the KLD based approach does not require adept knowledge of AE phenomena analysis as it does in the AE-hit based approach. The better results using KLD as the HI-constructing factor in comparison to AE hits can be explained by the fact that even though the AE hits have a better description of the AE activities’ nature, it is very difficult to extract the exact number because multiple events are happening at the same time, which leads to overlapping hits in the time domain signal. Through this process of hit detection, useful information can still be neglected, whereas KLD still allows extraction whilst retaining most of the useful information. In addition, the computational complexity is significantly less using KLD because it does not require extensive analysis for the detection threshold, which is of paramount importance in the AE-hit approach. From this test of RUL prognosis and the fitness evaluation, it can be concluded that KLD is reliable as a HI-constructing factor. There is still one problem, which is that, in a few cases, unlike the AE-hit based HI, the prognosis using KLD based HI outputs a result in which the expected useful life expiration comes after the actual date. Even though this is undesirable in the context of prediction, the error is small and will be investigated in future studies.

## 5. Conclusions

This paper presented an autonomous health indicator (HI) constructor for remaining useful lifetime (RUL) prognosis on concrete structures using Kullback–Leibler Divergence (KLD) with in-service acoustic emission (AE) monitoring. By using a reliable HI for the prognosis task, the user can be warned of future failure and, thus, maintenance can be performed accordingly to minimize safety risks and financial concerns. The process of HI construction was initialized from raw data, which was processed by the Fast Fourier Transform and fed to a constructor to generate the HI. The constructor was a deep neural network (DNN) structure, whose parameters were obtained through pretraining and fine-tuning with a stacked autoencoder (SAE). KLD values of the data were calculated between a reference normal condition and unknown condition in a one-second window, which were then utilized as the training label for the DNN. The KLD was chosen to portray the deterioration of concrete specimens because of its ability to describe how much a signal is different from another. As the deterioration of the concrete specimens worsens, more significant activities can be expected in the AE signal.

Afterward, evaluation of the constructed HI was presented to verify its reliability in the prognosis task for concrete specimens. The evaluation was divided into two categories: fitness analysis of the construction result and RUL prognosis using the constructed HI. Trendability and monotonicity were utilized as the metrics for fitness analysis, which showed a result of 0.68 and 0.69, respectively, comparable to the AE-hit based HI (at 0.68 and 0.68) and significantly better than other compared factors. A comparison was also performed in the RUL prognosis on the proposed HI and AE-hit based HI at two major time steps (350th and 450th) that vaguely marked the initialization of the micro and major fractures on the specimens. In the first prediction, the RUL predictions using three tests’ data were 28, 28, and 36, while in the second, they were 11, 18, and 16, respectively. The result of this comparison presented an outperformance of the proposed HI, especially on the second prediction. Through this evaluation, KLD was concluded as a reliable factor for HI construction.

In future research, the proposed HI construction can be expanded to not just reinforced concrete beams, but also possibly bridges, walls, and buildings, etc. Pre-processing is also expected to provide a better representation of the specimen’s health state. Other optimization techniques and structures will also be investigated in future work to create a better construction model. We will also aim to approach the HI construction from the system- and fault-specific aspect, to which the finite element method (FEM) simulation shall be implemented along with Failure Mode, Effects and Criticality Analysis (FMECA).

## Figures and Tables

**Table 1:** Fitness analysis of the proposed HI-constructing factor and other factors.

HI-Constructing Factor	Minimum Value	Maximum Value	Trendability	Monotonicity
**RMS**	0.0005	0.1529	0.347 ± 0.064	0.037 ± 0.022
**Kurtosis**	2.7496	5629.1	0.458 ± 0.050	0.004 ± 0.021
**Crest Factor**	3.0803	145.91	0.7416 ± 0.024	0.0013 ± 0.183
**Skewness**	−19.279	10.855	0.1077 ± 0.1020	0.0102 ± 0.0215
**Entropy**	0.0883	4.8324	0.1886 ± 0.1740	0.00196 ± 0.0234
**AE-hit**	0.0125	1	0.6801 ± 0.0489	0.6788 ± 0.079
**KLD**	0.0205	1	0.6807 ± 0.0873	0.6953 ± 0.0653

**Table 2:** RUL prognosis performance assessment of the proposed HI with AE-hit and AE-hit AHR HIs.

Test	HI-Constructing Factor	RUL Error Predicted at the 350th Time Step	RUL Error Predicted at the 450th Time Step
**α**	KLD	28 ± 7	11 ± 7
	AE-hit AHR	31 ± 5	19 ± 4
	AE-hit	32 ± 3	21 ± 4
**β**	KLD	28 ± 11	18 ± 7
	AE-hit AHR	37 ± 10	31 ± 7
	AE-hit	41 ± 7	34 ± 5
**γ**	KLD	36 ± 6	16 ± 5
	AE-hit AHR	38 ± 9	24 ± 3
	AE-hit	36 ± 3	24 ± 3

## Data Availability

The data is available upon request.
